# Segmenting Proteins into Tripeptides to Enhance Conformational Sampling with Monte Carlo Methods

**DOI:** 10.3390/molecules23020373

**Published:** 2018-02-09

**Authors:** Laurent Denarie, Ibrahim Al-Bluwi, Marc Vaisset, Thierry Siméon, Juan Cortés

**Affiliations:** LAAS-CNRS, Université de Toulouse, CNRS, 31400 Toulouse, France; ldenarie@gmail.com (L.D.); al.bluwi@gmail.com (I.A.-B.); marc@laas.fr (M.V.); nic@laas.fr (T.S.)

**Keywords:** conformational sampling, Monte Carlo, proteins, robotics-inspired approach

## Abstract

This paper presents an approach to enhance conformational sampling of proteins employing stochastic algorithms such as Monte Carlo (MC) methods. The approach is based on a mechanistic representation of proteins and on the application of methods originating from robotics. We outline the general ideas of our approach and detail how it can be applied to construct several MC move classes, all operating on a shared representation of the molecule and using a single mathematical solver. We showcase these sampling techniques on several types of proteins. Results show that combining several move classes, which can be easily implemented thanks to the proposed approach, significantly improves sampling efficiency.

## 1. Introduction

The study of proteins is essential to understanding living organisms, since they are responsible for most processes inside cells. Proteins are also of interest for other domains. For instance, proteins are pharmaceutical targets of drugs, their catalytic properties are exploited in biotechnology, and they are used as components of nano-devices in the rising field of bionanotechnology. A better understanding of the relationship between structural and dynamic features of proteins and their function is fundamental in all of these domains.

Protein investigations are very challenging because of the large size and flexibility of these biological molecules. X-ray crystallography [1] is the most widely-used experimental method to obtain atomic-scale data. However, it only provides a static picture. In this respect, nuclear magnetic resonance (NMR) spectroscopy is a more complete technique, since it is able to provide relevant structural and dynamic data of proteins in solution [2,3,4]. These data are nevertheless limited and averaged. Therefore, computational methods, which have been proposed over the last decades, are used as a complement to experimental methods to model proteins and to simulate their behavior. They are mostly based on molecular dynamics simulations or Monte Carlo methods [5,6], although alternative methods have been proposed in recent years [7,8], some of which are originating from robotics [9,10,11].

This paper presents an approach to enhance conformational exploration methods (note that a preliminary version of this work was presented at the ASME IDETC/CIE 2012 conference [12]). The approach is based on a mechanistic view of proteins [13]. The idea is to cut the protein into small fragments of three amino acid residues, which we refer to as *tripeptides*. Each fragment can be represented as a kinematic chain, similar to a robotic manipulator. Such a representation enables performing local deformations of the protein backbone model that preserve bond geometry, using closed-form inverse kinematics (IK) solvers [14]. This work focuses on the application of this approach for devising Monte Carlo move classes. Nevertheless, the tripeptide-based protein representation introduced below can be exploited within other types of methods, as further discussed in the conclusion.

The basic Monte Carlo (MC) method [6,15] explores the conformational space through a random walk. At each iteration, the protein conformation is randomly perturbed, and the trial move is accepted or rejected with a probability that depends on the potential energies of the old and the new states. The main difficulty involving the application of the MC method to proteins lies in devising suitable trial move classes for complex chain-like molecules. An effective move class would yield a good acceptance rate (therefore avoiding futile, expensive energy evaluations), while enabling the exploration of large regions of the conformational space. Along more than three decades, significant efforts have been devoted to the development of trial move classes to enhance the efficiency of MC methods applied to proteins and other chain-like polymers [16,17,18,19,20,21,22]. A comprehensive review was published by Vitalis and Pappu a few years ago [23]. Building on these important contributions, the approach presented in this paper enables devising and combining different types of move classes that can be easily implemented using a unique molecular representation and a single solver.

The paper presents the general aspects of the mechanistic protein representation using the tripeptide-based decomposition, and explains how to implement several move classes based on this representation. Note however that the application of this approach to structure prediction or other related problems is out of the scope of this paper. Our aim is to show the interest of the proposed tripeptide-based model to enhance protein backbone sampling.

## 2. Results and Discussion

### 2.1. Implemented Move Classes and Parameter Settings

The methods presented in this paper have been implemented in a software package called MoMA (for Molecular Motion Algorithms), which includes modeling tools and algorithms to sample conformations and transition paths of biomolecules. The open-source code of MoMA is not available yet. Nevertheless, binaries can be provided upon request.

**Figure 1:**
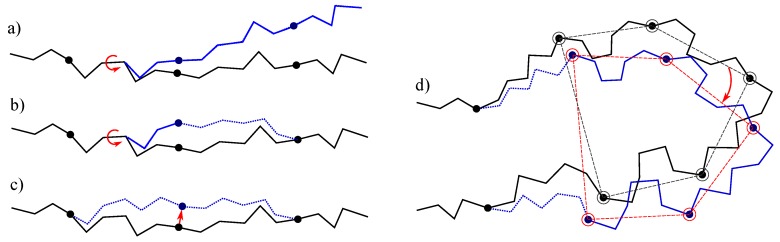
Illustration of the implemented move classes. (**a**) pivot move, which is the most frequently used move class within MC methods applied to chain-like molecules; (**b**) concerted rotation, as implemented using the proposed tripeptide-based model; (**c**) local move produced by the perturbation of the particle associated with one tripeptide; and (**d**) hinge move produced by a correlated perturbation of a set of particles. The protein backbone trace before and after the move are drawn in black and blue, respectively. Tripeptides that change their conformation are represented with dashed lines.

We have implemented four move classes. Three of them, producing fixed-end moves, are based on the proposed approach. The other one is simply based on the variation of a single bond torsion at each iteration. More precisely:

- The simplest class of trial moves, largely applied to sample the conformation of chain-like molecules, consists of perturbing a randomly selected bond torsion and then propagating the motion toward the end of the chain. Such moves, usually called pivot moves, are named here *OneTorsion* moves. They are illustrated in Figure 1a.
- The second move class is named *ConRot*, since it is inspired by the *concerted rotations* proposed by Dodd et al. [17]. It has been implemented using the proposed tripeptide-based model as follows: an amino-acid residue is randomly selected and one of its bond torsions (ϕ or ψ) is randomly perturbed; the backbone conformation of the next three residues (the next tripeptide) is modified by inverse kinematics in order to maintain fixed ends (see Section 3.2 for details). The move class is illustrated in Figure 1b.
- The third move class, called *OneParticle* moves, corresponds to the simplest move class involving tripeptide-based particle perturbations, as described in Section 3.2, and illustrated in Figure 1c.
- The last move class, called *Hinge* moves, corresponds to the rigid-body block moves described in Section 3.2, and illustrated in Figure 1d. The number of consecutive particles affected by the move is randomly sampled at each iteration between 3 and 10 (i.e., moves involve between 9 and 30 residues).

These four move classes have been applied within a basic MC method, using the Metropolis criterion [15] to accept or to reject trial moves. At each iteration, the algorithm randomly chooses between performing either a backbone move or a side-chain move. A side-chain move consists of randomly selecting a side-chain and perturbing all of its dihedral angles $\chi_{i}$. We have tested the four backbone move classes individually as well as a simple combination of all of them. The *Mixed* move class selects one of the four backbone move classes with equal probability at each iteration.

At each iteration of the MC method, the conformational parameters involved in the applied move class (bond torsions or oriented particle poses) are perturbed by adding a random value to their original value, sampled in the interval [−δ,δ]. Thus, the parameter δ defines the maximum perturbation step-size. For a meaningful comparative analysis, the values used for the perturbation of bond torsions ($\delta_{b}$), particle translations ($\delta_{pt}$) and particle rotations ($\delta_{pr}$) in each move class have to be chosen in such a way that they will produce average atom displacements of similar length. In general, this also implies that MC acceptance rates will be similar for all move classes. The values used in this work for the two test systems introduced below are presented in Table 1.

For energy evaluation, we use an in-house implementation of the AMBER parm96 force-field [24] with an implicit representation of the solvent using the Generalized Born (GB) approximation. A geometric filter is applied before energy evaluation with the aim of improving computational efficiency (note that our implementation of the energy force-field is not optimal in terms of computing time.). After applying each trial move, the model is checked for atom overlaps. A trial move is rejected if the distance between two non-bonded atoms is less than 70% of the van der Waals equilibrium distance [25]. In addition, for *ConRot*, *OneParticle* and *Hinge* move classes, a trial move is rejected if the IK solver fails to find a solution. All the tests have been performed at a temperature of 300 K.

### 2.2. Test Systems

**Figure 2:**
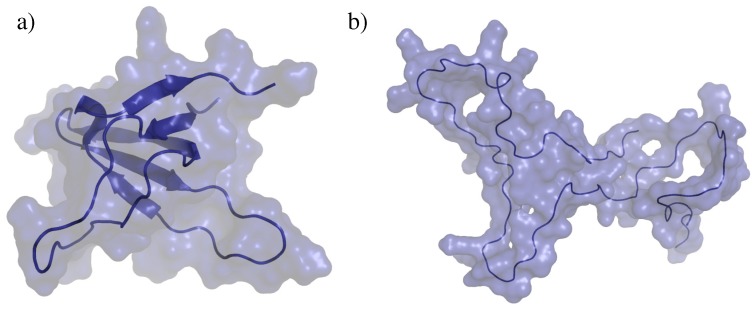
Proteins used for the analysis of the methods: (**a**) SH3 domain; (**b**) Sic1 protein.

We have chosen two different types of proteins to evaluate the performance of the move classes. The first one is a *SH3 domain* [26]. More precisely, we have used the SH3 domain of obscurin, represented in Figure 2a. This is a small globular protein composed of 68 amino-acid residues. It presents a relatively rigid beta-barrel-like core and two flexible loops. Its crystal structure is available in the Protein Data Bank (PDB ID: 1V1C). The second test system is an intrinsically disordered protein (IDP). More precisely, we have considered a disordered region of the *Sic1 protein* [27], containing 77 residues. A model of this protein region is shown in Figure 2b. The model was generated using the Flexible-Meccano method [28] for sampling a statistically probable backbone conformation, and SCWRL4 [29] for placing the side-chains. Note that, although the application of MC methods to sample the extremely vast conformational space of IDPs may appear unrealistic, it can be interesting for more specific problems such as the analysis of the energy landscape around a biologically functional conformation of the protein (e.g., a conformation of the IDP while interacting with another molecule).

The two structures were locally energy-minimized before running the tests. Starting from these initial conformations, the MC method was iterated until the generation of $2\times 10^{6}$ accepted samples for each molecule, using the four move classes individually or combined together. Each test was repeated three times. All tests using the same settings provided similar results.

The results presented in this paper are not aimed at providing new insights into these biological systems, but to serve as a proof of concept and to show the interest of the proposed approach for different types of proteins.

### 2.3. Computational Performance

Table 2 contains results on the computational performance of the methods, averaged over the 3 runs. It shows the MC acceptance rate for backbone moves, the total number of iterations (considering backbone and side-chain moves) and the overall CPU time for each system and move class. Tests were run on a single Intel^®^ Xeon^®^ E5-1650 processor at 3.2 GHz. These results show that, for a similar MC acceptance rate, generating a given number of samples ($2\times 10^{6}$ in this case) requires more computational resources using the *OneTorsion* move class compared to the other move classes, which apply fixed-end motions. The reason is that, although the *OneTorsion* move class does not require solving inverse kinematics, a significant amount of computing time is needed for propagating atom motions along the chain by *forward kinematics* and to recompute interaction energies between atom pairs. In other words, the computing time needed by the *ConRot*, *OneParticle* and *Hinge* move classes to solve inverse kinematics is largely compensated since only the positions of a small number of atoms need to be updated after each move, which also decreases the cost of energy recalculation. Nevertheless, computing time is probably not the most important indicator of the performance of the different move classes. Indeed, the numbers in Table 2 have to be analyzed together with other data related to the quality of the sampling strategy in terms of energy and distance distributions, as discussed below.

### 2.4. Distribution of Sampled States

**Figure 3:**
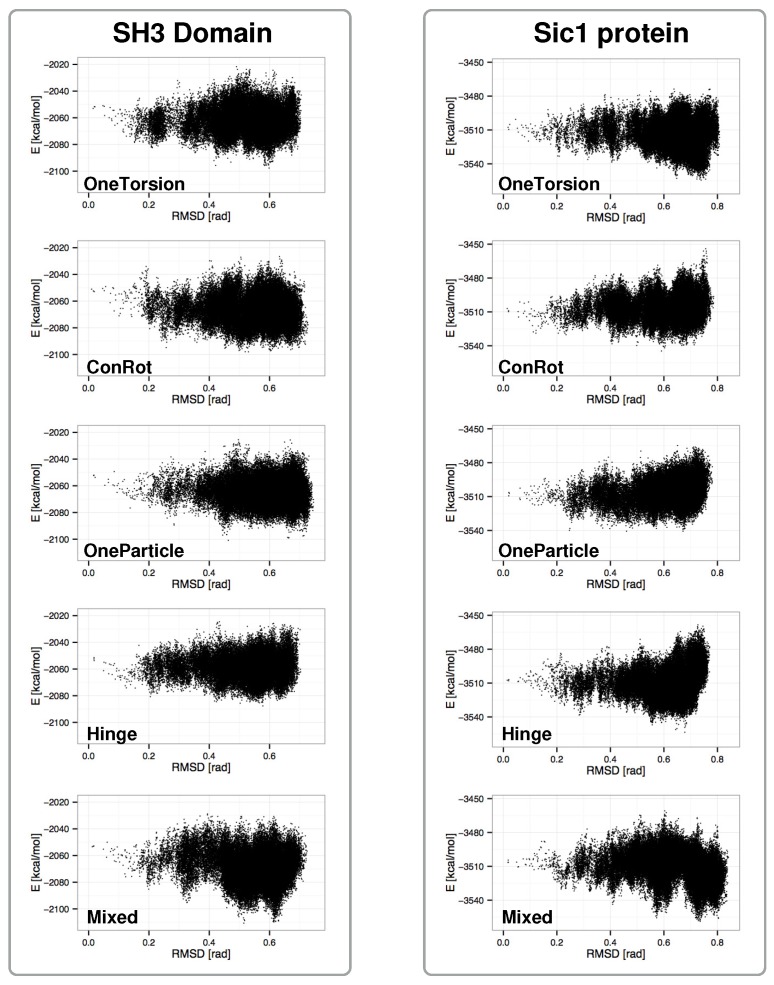
Projection of sampled states on distance vs. energy plots for the SH3 domain and the Sic1 protein.

Figure 3 shows plots aimed at comparing the performance of the different move classes in terms of conformational space coverage for the SH3 domain and the Sic1 protein. They represent the projection of the sampled states on two dimensions: the distance with respect to the initial structure and the potential energy. The distance is measured as the root mean square deviation (RMSD) of the bond torsion angles from the initial conformation. For clarity reasons, only one in every one hundred sampled conformations has been plotted (i.e., 20,000 samples from each run). Each plot includes the results of the three runs.

Several conclusions can be extracted from the analysis of these plots: (1) the *Mixed* move class shows the best performance. The combination of move-classes provides samples that have lower energies, and conformational coverage (distances from a reference conformation) is comparable or better than for the individual move classes; (2) move classes involving a larger number of atoms, i.e., *OneTorsion* and *Hinge*, show better performance on the disordered Sic1 protein, which presents higher conformational variability than the globular SH3 domain. For the Sic1 protein, these move classes reach lower-energy regions compared to *ConRot* and *OneParticle*; (3) as it could be expected, the performance of *ConRot* and *OneParticle* moves is very similar for both test systems; and (4) the *Hinge* move class shows slightly poorer exploration capabilities compared to the other move classes. The maximum distances of the samples to the reference conformation are smaller when using this move class alone. The difference is more significant for the SH3 domain.

### 2.5. Exploration Efficiency Analysis

The distance vs. energy plots permit comparing the different move classes in terms of coverage, but they do not allow us to quantitatively evaluate their exploration efficiency. Furthermore, these plots illustrate the performance at the end of a long MC run, but do not show the short-term exploration capabilities of the different move classes, which are of interest for applications where MC methods are run for a short time, such as structural relaxation. This section presents additional tests allowing such an analysis.

#### 2.5.1. Time Dependent RMSD Function

The time dependent RMSD function, rmsd(τ), is aimed at showing the rapidity of the exploration process. It represents the average distance between conformations separated by τ MC steps:

$$\mathrm{rmsd}\left(\tau \right)={\langle \mathrm{RMSD}(q_{k},q_{k+\tau })\rangle }_{k},$$

where $q_{k}$ is the configuration of the system after the *k*th trial in the MC simulation, and ${\langle X(k)\rangle }_{k}$ denotes the average of the observable *X* over all *k*.

**Figure 4:**
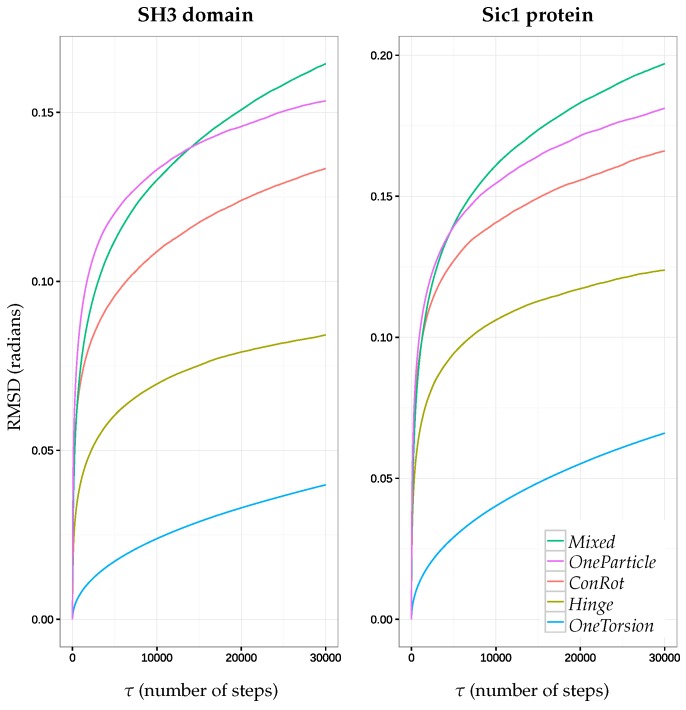
Plots of the function rmsd(τ) for each move class during MC runs for the SH3 domain (**left**) and the Sic1 protein (**right**).

Figure 4 shows the time dependent RMSD function for each move class and for the two molecules, using the results from the aforementioned MC simulations ($2\times 10^{6}$ accepted samples), for τ values up to 30,000 MC steps. It provides interesting additional information with respect to the analysis in the previous section, and allows for highlighting more significant differences between the move classes: (1) it confirms that the *Mixed* move class performs better than the other individual move classes; (2) although the *OneParticle* and *ConRot* move classes perform very similarly, it appears that the *OneParticle* move class explores a bit faster than *ConRot*. In the case of the SH3 domain, *OneParticle* can even compete with the *Mixed* move class on small time scales; and (3) the *Hinge* move class performs almost two times worse than the *Mixed* move class for both proteins, while the *OneTorsion* move class performs almost four times worse. This last result, together with results in the previous section, show that, although *OneTorsion* moves may provide good performance in terms of coverage (particularly for the disordered Sic1 protein), convergence can be slow.

#### 2.5.2. Autocorrelation Time

Autocorrelation is a statistical tool that computes the correlation of a time series with a lagged version of itself. In the context of a MC simulation, it is used to characterize how new accepted states gradually become independent from the previous ones. Formally, the autocorrelation of an observable *O* with a lag τ is defined by:

$$\mathrm{acf}\left(\tau \right)=\frac{E[\left(O_{k}-\langle O\rangle \right)\left(O_{k+\tau }-\langle O\rangle \right)]}{\mathrm{Var}(O)},$$

where E[X] denotes the expected value of *X*, Var(O) denotes the variance of *O*. $O_{k}$ and $O_{k+\tau }$ represent two series of observations of *O* during the MC run with a lag of τ steps. This formula only holds true when the simulation is stationary. In the context of MC, this requirement implies that the simulation is long enough for the autocorrelation value to converge. Since this would require very long computing time for large systems such as the SH3 domain or the Sic1 protein, we have used a smaller system for this test. Inspired by related work [22,30], we have used a 14-alanine molecule with a structural constraint: starting from a low energy stable state, corresponding to an α-helical conformation, the two end residues were blocked in position and orientation so that the system could only fluctuate around this state. This constraint, associated with the small size of the system, restricts the applicable move classes to *ConRot*, *OneParticle*, and a *Mixed* move class involving both of them.

**Figure 5:**
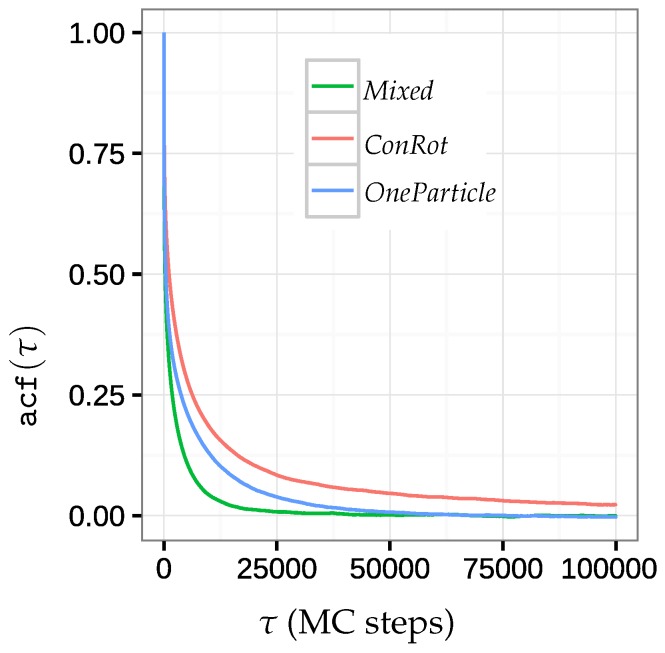
Plots of the average autocorrelation function over the 20 central dihedral angles of 14-alanine for the *ConRot*, *OneParticle*, and *Mixed* move classes.

Starting from the helical conformation, the MC method was iterated until $10^{8}$ samples were accepted for the two move classes individually or combined together (no side-chain moves were performed). Figure 5 shows the average autocorrelation function over the 20 central dihedral angles for each move class. We can observe that the *Mixed* move class yields the fastest independence time, whereas the autocorrelation of *ConRot* seems to stagnate when approaching zero.

For a more precise evaluation, we computed the characteristic autocorrelation time, also called the integral autocorrelation time. It is defined by:

$$\tau_{int}=\frac{1}{2}+\sum_{\tau =1}^{\infty }\mathrm{acf}\left(\tau \right).$$

**Figure 6:**
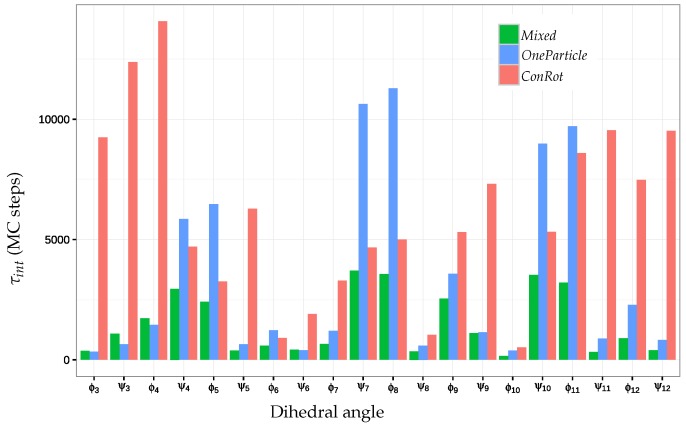
Autocorrelation times $\tau_{int}$ (in MC steps) of the 20 central dihedral angles of 14-alanine for the *ConRot*, *OneParticle*, and *Mixed* move classes.

In practice, to reduce computational cost and noisy values, the summation is usually truncated. We have summed for all $\tau \leq 5\tau_{int}$. The autocorrelation times of the 20 central dihedral angles are plotted in Figure 6, and the statistics for each move class are summarized in Table 3. These results allow us to quantify the improvement of autocorrelation time between the different move classes. The *OneParticle* move class has an autocorrelation time about 1.8 times lower on average than the *ConRot* move class, while the *Mixed* move class consistently outperforms the *OneParticle* move class by a factor of 2. These results confirm that the *Mixed* move class is much more efficient than the individual move classes.

### 2.6. Additional Results for Ubiquitin

**Figure 7:**
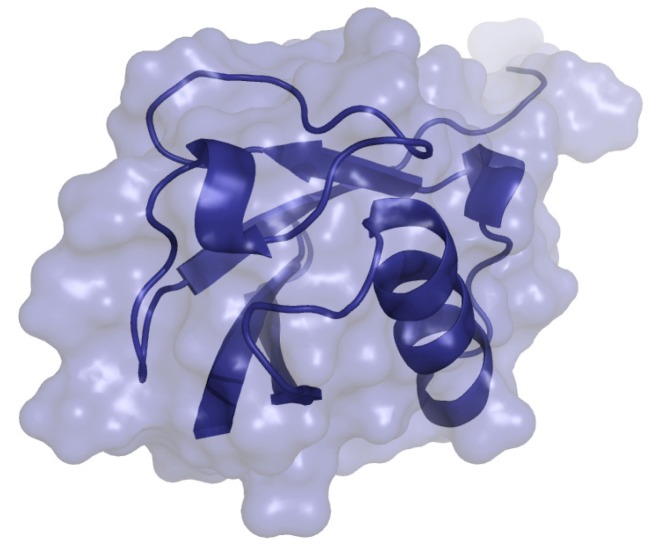
Structure of ubiquitin.

We performed additional tests on human ubiquitin, a well studied system with experimental and computational methods [31]. The structure of this protein (PDB ID: 1UBQ) is shown in Figure 7. Our main goal was to compare, qualitatively, the results obtained with the methods proposed in this paper to those presented by Bottaro et al. [22]. The authors of this related work propose an extension of the concerted rotation move class, called CRISP (Concerted Rotations Involving Self-consistent Proposals), and use ubiquitin to demonstrate the ability of their enhanced MC method to reproduce structural variations around the native conformation, with a performance comparable to molecular dynamics simulations. Note that the CRISP move class is compatible with the approach presented in this paper, and could be implemented using the proposed framework.

For the comparative analysis, we applied the *ConRot* and *OneParticle* move classes, as well as a *Mixed* move class involving both of them. Three MC simulations of $15\times 10^{6}$ steps each were performed for each move class, starting from a relaxed state of the protein. The plots presented below display average values of the three runs. Each MC simulation randomly alternates between the compared move class (probability of 20%), a side-chain perturbation (probability of 75%), or a *OneTorsion* move (probability of 5%). We used the same step sizes than for the SH3 domain (see Table 1).

**Figure 8:**
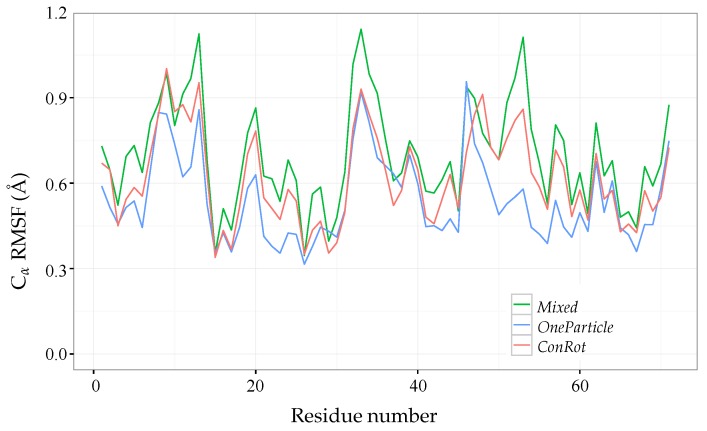
Ubiquitin C${}_{\alpha }$ root mean squared fluctuations (RMSF) from $15\times 10^{6}$ steps of MC simulations using *ConRot*, *OneParticle*, and *Mixed* move classes.

Figure 8 shows the root mean squared fluctuations (RMSF) of the C${}_{\alpha }$ atoms. The RMSF is calculated as the RMSD from the mean (equilibrium) position. As for the previous experiments, the figure shows that the *ConRot* and *OneParticle* move classes perform similarly, and that the *Mixed* move class explores more widely than the individual move classes. In this case, *ConRot* slightly outperforms *OneParticle*. This can be explained by the compactness of the ubiquitin structure with respect to the SH3 domain and the Sic1 protein, which favours local moves involving few atoms. Interestingly, although we are using a different force-field and a less sophisticated sampling method for the side-chains, the C${}_{\alpha }$ RMSF profiles are very similar to those presented in the referred publication by Bottaro et al. [22].

**Figure 9:**
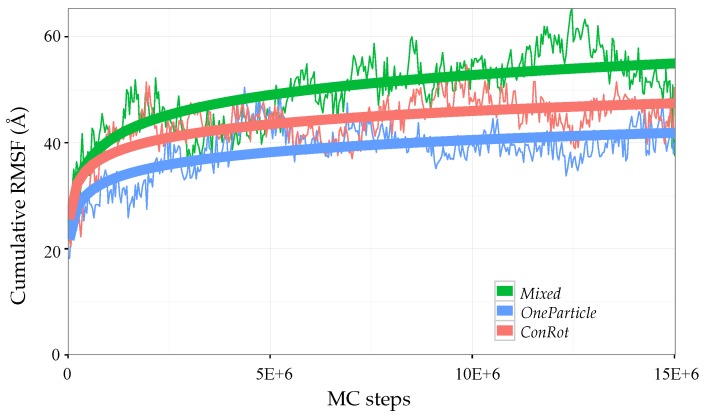
Evolution of the cumulative C${}_{\alpha }$ root mean squared fluctuations (RMSF) of ubiquitin with the number of MC steps using *ConRot*, *OneParticle*, and *Mixed* move classes.

The efficiency of the different move classes can be roughly evaluated and compared by looking at the evolution of the RMSF with the number of iterations. The RMSF of a particular atom is expected to grow with the simulation time, and eventually converge to a maximum value when the energy basing around the equilibrium state has been completely explored. Figure 9 shows the evolution of the cumulative RMSF (i.e., the sum of the RMSF values of all C${}_{\alpha }$ atoms) with the length of the simulation. The plot confirms a faster exploration of the *Mixed* move class, which allows reaching a cumulative RMSF value around 50 Å after $15\times 10^{6}$ MC steps, whereas the individual *ConRot* and *OneParticle* move classes only attain 40 Å. These values are also very similar to the ones obtained by Bottaro et al. [22]. In fact, a direct comparison of Figure 9 in this paper and Figure 4 in the referred publication suggests a faster convergence of our *Mixed* move class with respect to the CRISP move class, which requires around 10 times more iterations to reach 50 Å ($5\times 10^{7}$ MC steps instead of $5\times 10^{6}$ MC steps). However, such a direct comparison should be put into perspective because the models and the implementations are different.

## 3. Materials and Methods

### 3.1. Protein Model

#### 3.1.1. Mechanistic Model

The conformation (i.e., spatial arrangement) of a protein can be defined by the *Cartesian coordinates* of all its constituent atoms, or by a vector of *internal coordinates* that represent the relative position of bonded atoms. These internal coordinates correspond to the bond lengths, bond angles and bond torsions. Since the bond lengths and bond angles vary very slightly at room temperature, they are often considered to be constant parameters in molecular simulations [32]. Under such assumptions, the bond torsions are the only degrees of freedom of the molecule. An additional simplification of molecular models is to consider that double bonds, such as peptide bonds in proteins, are rigid connections (i.e., the dihedral angle ω associated with the peptide bond torsion is constant). In summary, the variable parameters that define the conformation of a protein backbone are the pairs of dihedral angles, ϕ and ψ, of all its constituent amino-acid residues. The conformation of the side-chains is determined by a variable number of dihedral angles $\chi_{i}$ for each residue.

In this work, we consider the aforementioned rigid geometry assumption. Nevertheless, small variations of bond lengths, bond angles and peptide bond torsions, which can be important from a structural point of view [33], can be considered within our approach, as discussed below.

Using the internal coordinate representation described above, proteins can be modeled as articulated mechanisms. The bodies of the mechanism correspond to rigidly-bonded atom groups, and the joints are the bond torsions. The kinematic chains corresponding to the protein backbone and side-chains can then be modeled using standard conventions usually applied in robotics. In this work, we have used the *modified Denavit–Hartenberg* (mDH) convention [34]. Following this convention, a Cartesian coordinate system $F_{i}$ is attached to each rigid atom group. The relative location of consecutive frames in a kinematic chain can be then defined by a homogeneous transformation matrix of the form:

$${}^{i-1}T_{i}=\left(\begin{array}{cccc} \cos\theta_{i} & -\sin\theta_{i} & 0 & a_{i-1}\\ \sin\theta_{i}\cos\alpha_{i-1} & \cos\theta_{i}\cos\alpha_{i-1} & -\sin\alpha_{i-1} & -d_{i}\sin\alpha_{i-1}\\ \sin\theta_{i}\sin\alpha_{i-1} & \cos\theta_{i}\sin\alpha_{i-1} & \cos\alpha_{i-1} & d_{i}\cos\alpha_{i-1}\\ 0 & 0 & 0 & 1. \end{array}\right)$$

**Figure 10:**
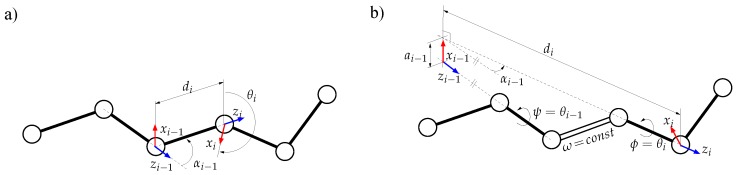
Geometric model of the protein backbone: (**a**) in a general case; (**b**) in the particular case of a rigid peptide bond. In the general case, the mDH parameters involved in the homogeneous transformation matrices between consecutive atom groups are obtained directly from bond lengths and bond angles. In the case of a rigid peptide bond, this requires some (simple) geometric operations. Note that the origin of a frame attached to an atom group does not necessarily correspond to an atom position.

The elements of ${}^{i-1}T_{i}$ depend on the bond geometry. Assuming constant bond lengths and bond angles, the parameters $d_{i}$ and $\alpha_{i-1}$ (which can be directly computed from them) are constant. The bond torsion angle $\theta_{i}$ is the only variable parameter. Figure 10 illustrates the method to assign the frames and to obtain the mDH parameters in a general case, and when peptide bond torsion angles (ω) are considered to have an arbitrary constant value. Note that, in the general case, when all the bond torsion angles are variable, the parameters $a_{i}=0$.

#### 3.1.2. Decomposition into Tripeptides

**Figure 11:**
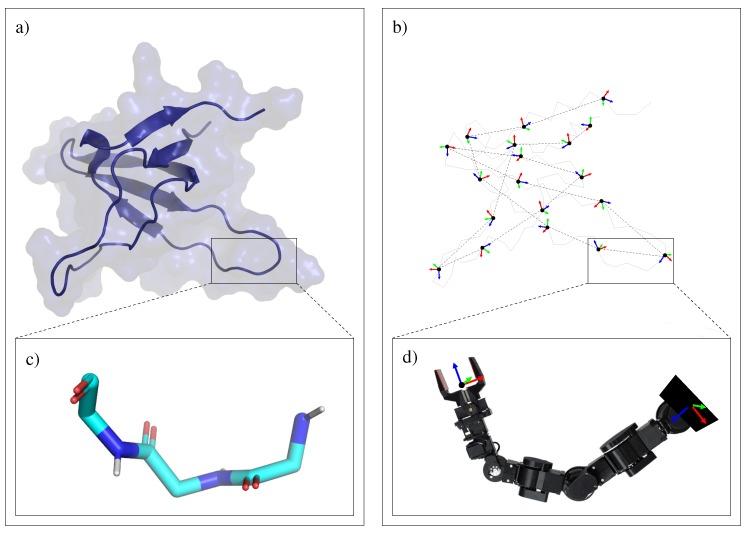
Illustration of the protein subdivision approach. Fragments of three amino-acid residues are treated as kinematic chains, similar to robotic manipulators.

The main idea proposed in this paper is to segment the protein chain into fragments of three amino acid residues, which we refer to as *tripeptides*. The reason for choosing such a subdivision is that each tripeptide backbone involves six degrees of freedom (three pairs of ϕ, ψ angles), corresponding to the shortest fragment with full instantaneous mobility of the end-frame relatively to the base-frame (i.e., the relative position and orientation of the two frames can change in any direction). Figure 11 illustrates this idea. Figure 11a shows a protein model with a ribbon representing the backbone embedded in the model of the protein surface. Figure 11b represents the protein backbone trace with the frames corresponding to the connections between consecutive tripeptides. Figure 11c,d represent the chemical and the mechanistic models of the backbone of a single tripeptide, respectively. As depicted in the figure, the tripeptide backbone can be seen as a robotic manipulator with six revolute joints. The base of the manipulator corresponds to the first body of the tripeptide backbone (i.e., the first rigid atom group in the backbone of the first amino-acid residue). Since tripeptides are linked through rigid peptide bonds, the location of the end effector of tripeptide *i* can be determined from the base-frame of tripeptide i+1 by a constant transformation. Given the location of the base-frame and the end-frame, the conformation of a tripeptide backbone can be determined by efficient analytical *inverse kinematics* (IK). The IK problem formulation and the particular solver applied in this work are described in the next section. Consequently, the conformation of the whole protein backbone can be determined from the pose of a single reference frame for each tripeptide, the one attached to the first body of each tripeptide backbone. In the following, we will refer to these reference frames as (oriented) *particles*. The last affirmation is true for all the protein backbone except two short fragments at the N-terminal and C-terminal ends of the chain. Since the choice of the first residue for the decomposition into tripeptides is arbitrary (and may change during the conformational exploration process), the polypeptide chain model involves two terminal fragments, containing up to three residues, which require a particular treatment. The conformations of these terminal fragments are directly defined by their internal bond torsions.

### 3.2. Devising Move Classes

This section presents a unified approach for devising different move classes that can be employed in the context of a Monte Carlo framework. These classes all utilize the tripeptide-based representations described above. The principle consists of perturbing the pose (position and orientation) of a set of particles, and then adapting the conformation of several tripeptides in order to keep the integrity of the molecular chain while maintaining the local geometry of the bonds (i.e., constant bond lengths and bond angles). Several strategies can be considered for perturbing the pose of particles. The number of particles selected for perturbation and the correlation/uncorrelation between the motion direction of several particles leads to different move classes, more or less local, and more or less collective.

Next, we explain how to implement simple general-purpose move classes. Here, we only deal with unbiased random moves. However, our approach is also suitable for devising biased moves. In such a case, the selection of the particles to be perturbed and the motion directions would be determined depending on the specific context. For instance, moves could be devised to deform proteins while simulating the interaction with other molecules, or for applications such as all-atom model fitting into lower-resolution electron density maps.

The move classes presented below, as well as other ones such as *pivot moves* applied to a single bond torsion, can be combined within a higher-level sampling protocol that selects a move class at each iteration. In addition, the constraints on bond lengths, bond angles, and peptide bond torsions imposed by the tripeptide-based model can be relaxed. This can be done by performing separate MC moves on these parameters, or by slightly perturbing the geometry of some or all the bonds in a tripeptide before applying the IK solver. Note that a similar approach to include variable bond geometry has been applied in related work [35].

Side-chain moves, which are usually performed separately from backbone moves, are not treated in this section. Side-chain conformations can be sampled by simple perturbations of the bond torsion angles $\chi_{i}$ or following more sophisticated approaches [19,36]. Moreover, different strategies can be adopted to combine backbone and side-chain trial moves in a suitable manner [37].

#### 3.2.1. Perturbing Particles

Different move classes can be easily implemented from the proposed tripeptide-based model. As an example, three move classes involving perturbations of one or several particles as explained next. As explained in Section 2.1, we have implemented several of them as well as other frequently-used moves for an empirical evaluation.

One-particle moves: Local moves can be implemented by perturbing the pose of a single particle, as depicted in Figure 1c. Such perturbation implies that the two tripeptides linked through this particle (i.e., with end-frame or base-frame defined from it) change their backbone conformation. In other words, 12 consecutive bond torsions are modified, while the rest of the protein conformation remains unchanged. This type of moves has a similar effect to other local, fixed-end move classes [17,18,38] proposed since the seminal work of Gō and Scheraga [16].

Flexible fragment moves: The previous move class can be extended to larger fragments by applying perturbations to a set of *n* consecutive particles. If the particles are perturbed independently (i.e., in different random directions), the backbone of n+1 tripeptides is affected by the move. This move class has a similar effect to moves based on the cyclic coordinate descent method (CCD) [39]. Such moves consist of breaking the chain at an arbitrary point, performing a random perturbation of several bond torsions at one of the sides, and applying CCD to close the chain again (but with a different, perturbed conformation). An interesting advantage of the proposed move class with respect to CCD-based moves is that the deformation of sub-fragments can be easily modulated (e.g., larger perturbations for the middle particles in the fragment and smaller perturbations near the ends).

Rigid-body block moves: A simple variant of the previous move class may produce a very different effect, as illustrated in Figure 1d. In this case, *n* consecutive particles are also perturbed, but the perturbations are correlated in such a way that the particles do not move with respect to each other. Indeed, the perturbation is applied to a fictitious rigid body formed by the set of *n* particles. In principle, a random translation and rotation around an arbitrary axis could be tried. Nevertheless, it may be more interesting to apply moves that simulate hinge motions. Note that only two “hinge” tripeptides (i.e., the tripeptides preceding and following the selected particle sequence) change their conformation. Hinge-like moves, called Closed Rigid-body Rotation Under Bond-angle Restraints (CRRUBAR) moves [20], have been shown to be particularly efficient for sampling conformations of proteins. Although the proposed method involves more complex algebraic operations than CRRUBAR, it presents the advantage that bond angles do not need to be distorted.

#### 3.2.2. Solving Inverse Kinematics for a Tripeptide

Once the location of the particles is set, obtaining the conformation of each tripeptide requires solving an inverse kinematics (IK) problem for the chain corresponding to its backbone. As explained above, the model of a tripeptide backbone is similar to a six-revolute (6R) serial manipulator with general geometry. Its conformation is defined by the vector of six angles $\left\{\theta_{1},\theta_{2},\theta_{3},\theta_{4},\theta_{5},\theta_{6}\right\}$, corresponding to the three pairs of ϕ, ψ bond torsion angles. The IK problem is to find values for this vector of angles such that they solve the following matrix equation: ${}^{0}T_{1}{\;}^{1}T_{2}{\;}^{2}T_{3}{\;}^{3}T_{4}{\;}^{4}T_{5}{\;}^{5}T_{6}=T_{rel}$, where $T_{rel}$ is the homogeneous transformation matrix representing the relative pose of two consecutive particles, and each ${}^{i-1}T_{i}$ is the transformation matrix between consecutive atom groups in the chain, which is a function of $\theta_{i}$.

The method applied in this work for solving the IK problem for a general 6R serial kinematic chain has been adapted from the solver proposed by Renaud [40,41]. This solver is based on algebraic elimination theory, and develops an ad hoc resultant formulation inspired by the work of Lee and Liang [42,43]. Starting from the matrix equation representing the IK problem, the elimination procedure leads to an 8-by-8 quadratic polynomial matrix in one variable. The problem can then be treated as a generalized eigenvalue problem, as was previously proposed by Manocha and Canny [44], for which efficient and robust solutions are available [45]. Our implementation applies the Schur factorization from the linear algebra package LAPACK [46]. Further details on the applied IK solver are provided in a technical report by Renaud [41].

This solver has been successfully applied in previous works on protein and polymer modeling [47,48]. The advantage of this semi-analytical method with respect to numerical (optimization-based) methods, such as CCD, is that it provides the exact solution in a single iteration, not suffering from slow convergence issues. The solver is very computationally efficient, requiring about 0.2 milliseconds on a single processor. Note, however, that our approach is not dependent on this solver, so that other IK methods [17,44,49,50] could be applied.

In general, the IK problem for a 6R serial kinematic chain has a finite number of solutions (up to 16 in the most general case). All the solutions correspond to geometrically valid conformations of the tripeptide backbone with fixed ends defined by the pose of the particles. Depending on the type of application, several strategies can be adopted to select one of the solutions. The simplest strategy within a MC method is to select one at random. However, if detailed balance needs to be satisfied for a correct sampling of equilibrium fluctuations in the canonical ensemble, some works [17,19,30,49] recommend to take one of the solutions with a probability that depends on the Boltzmann factor and another term, called the Jacobian, which attempts to correct for the non-uniformity in the distribution of the torsion angles introduced by the fixed-end moves. Alternatively, when the moves are performed for finding energy minima, all the conformations can be evaluated in order to keep the best one (in terms of the Boltzmann factor, for instance). Otherwise, when the goal is to simulate continuous motions, the closest conformation to the one prior to the perturbation is selected in order to minimize the jumps in conformational space (if none of the solutions remains within a distance threshold that depends on the perturbation step-size, the local move is rejected). Note that this strategy obeys detained balance if a “reverse proximity criterion” is satisfied [51].

## 4. Conclusions

We have presented a unified approach to devise efficient Monte Carlo move classes for chain-like molecules. Based on a subdivision of the protein into tripeptides and on the application of efficient 6R inverse kinematics, many of the move classes that have been proposed in the last decades to enhance protein backbone sampling can be easily implemented. The approach is general, being applicable to proteins on any size, shape and topology. First results for proteins from different structural classes (globular and disordered) have been presented as a proof of concept. The overall conclusion of these results is that mixing move classes provides better results than using a single move class, as also suggested by related work on MC methods. Combining different move classes is a straightforward task following the proposed approach.

In this paper, we have only presented results of simple, unbiased MC sampling. However, the approach could be implemented within more advanced methods for conformational sampling [52] or global optimization [53]. Future work could also involve the implementation of a more sophisticated sampling strategy performing different types of trial moves selected among a variety of parameterized move classes.

We also envisage investigating the application of our approach to enhance other types of conformational exploration methods. More precisely, we plan to introduce the tripeptide-based representation within path-planning-based methods to compute motions associated with protein–ligand interactions [54]. Other research directions would concern the development of methods to refine predictions of protein–protein docking methods or to fit structures into electron density maps from cryo-electron microscopy (cryo-EM) using flexible protein models. For these applications, the proposed tripeptide-based representation is particularly interesting since it enables a very simple implementation of biased moves that deform regions of the protein with respect to interactions with other molecules or to locally improve structure fitting. Computational protein design is another potential application area for the proposed approach. It has been shown that introducing local backbone perturbation may significantly improve the results provided by protein design methods [55]. Using our approach, different classes of local moves around mutated residues can be easily implemented inside stochastic algorithms for protein design.

## Figures and Tables

**Table 1:** Perturbation step-sizes.

	*OneTorsion*	*ConRot*	*OneParticle*		*Hinge*
	$\delta_{b}$	$\delta_{b}$	$\delta_{\mathrm{pt}}$	$\delta_{\mathrm{pr}}$	$\delta_{\mathrm{pr}}$
**SH3 domain**	0.01 rad	0.025 rad	0.05 Å	0.003 rad	0.01 rad
**Sic1 protein**	0.02 rad	0.025 rad	0.05 Å	0.003 rad	0.02 rad

**Table 2:** Computational performance.

	Move Class	Acc. Rate	# Iterations	T${}_{\mathrm{CPU}}$
**SH3 domain**	*OneTorsion*	0.68	$3.28\times 10^{6}$	63 h
	*ConRot*	0.56	$3.55\times 10^{6}$	51 h
	*OneParticle*	0.42	$4.06\times 10^{6}$	56 h
	*Hinge*	0.59	$3.46\times 10^{6}$	57 h
	*Mixed*	0.56	$3.58\times 10^{6}$	57 h
**Sic1 protein**	*OneTorsion*	0.56	$3.54\times 10^{6}$	89 h
	*ConRot*	0.65	$3.25\times 10^{6}$	63 h
	*OneParticle*	0.52	$3.66\times 10^{6}$	69 h
	*Hinge*	0.53	$3.60\times 10^{6}$	75 h
	*Mixed*	0.57	$3.49\times 10^{6}$	74 h

**Table 3:** Autocorrelation times $\tau_{int}$ (in MC steps) of the *ConRot*, *OneTorsion*, and *Mixed* move classes.

Move Class	Average	Min	Median	Max
*ConRot*	6016	512	5315	14,070
*OneParticle*	3426	343	1215	11,281
*Mixed*	1518	157	985	3706
